# The irrelevancy of game-type in the acquisition, development, and maintenance of problem gambling

**DOI:** 10.3389/fpsyg.2012.00621

**Published:** 2013-01-17

**Authors:** Mark D. Griffiths, Michael Auer

**Affiliations:** Psychology Division, International Gaming Research Unit, Nottingham Trent UniversityNottingham, UK

Although most people gamble occasionally for fun and pleasure, gambling brings with it inherent risks of personal and social harm. According to the most recent British Gambling Prevalence Survey there are ~350,000 problem gamblers in the UK which equates to just under 1% of the adult population (Wardle et al., 2011). Problem and pathological gambling can negatively affect significant areas of a person's life, including their physical and mental health, employment, finances, and interpersonal relationships (e.g., family members, financial dependents) (Griffiths, 2004). Furthermore, there are significant co-morbidities with problem gambling, including depression, alcoholism, and obsessive-compulsive behaviors (Petry et al., 2005; Desai and Potenza, 2008). These co-morbidities may exacerbate, or be exacerbated by, problem and pathological gambling. The availability of opportunities to gamble and the incidence of problem gambling within a community are also known to be linked (e.g., Pearce et al., 2008).

Anyone coming into the gambling studies field from a psychological perspective would probably conclude from reading the literature that problem and pathological gambling is associated with particular game types. More specifically, there appears to be a line of thinking in the gambling studies field that casino-type games (and particularly slot machines) are more likely to be associated with problem gambling than lottery-type games (Griffiths, 1994; Meyer et al., 2009).

In this opinion paper it is argued that when it comes to problem gambling, game-type is irrelevant and that the most important factors along with individual susceptibility and risk factors of the individual gambler (which are not discussed here and beyond this paper's remit), are the structural characteristics relating to the speed and frequency of the game (and more specifically event frequency, bet frequency, event duration, and payout interval) rather than the type of game. It is also argued that researchers in the gambling studies field need to think about game parameters rather than game type when it comes to any association with problem and pathological gambling.

## Event frequency and bet frequency

Griffiths (1993); Parke and Griffiths (2007) notes that event frequency refers to the number of events that are available for betting and gambling within any given time period. For example, a lottery draw may occur once a week but a slot machine may allow 15 chances to gamble inside 1 min. In this example, slot machine gambling has a higher event frequency than lottery gambling. Bet frequency refers to the number of bets or gambles placed in any given time period. Using lottery playing as example, Parke and Griffiths (2007) note that multiple tickets (e.g., 10 tickets) can usually be purchased as frequently as desired before any single lottery draw. In this instance, bet frequency would be equal to 10 but event frequency would be equal to 1. Therefore, event frequency can often be much lower than bet frequency and it is possible for players to spend more than they can afford even with a low event frequency.

Further empirical research is needed into the relationship between event frequency and bet frequency (Parke and Griffiths, 2007). This is because researchers often assume that event frequency and bet frequency have a strong relationship (i.e., the higher number of betting/gambling events—the higher the frequency of betting/gambling). However, this may not be the case. As Parke and Griffiths (2007) have noted:

“*Although, players can place many bets on just one gambling event, the outcome of this event can influence future betting activity. By outcomes, we are essentially referring to winning or losing. Losing can often create financial and emotional motivation to continue betting i.e. chasing … It could be speculated that the satisfaction from winning may reduce motivation for further betting in the short-term, or it may increase betting as a result of increased bankroll, illusions of control and/or cognitive biases. Therefore, a higher event frequency not only offers more opportunity and choice for betting, but also affects motivation for betting through revealing consequential wins and losses at the end of each event*” (p. 226).

## Event duration

Another important gaming parameter is event duration. This refers to how fast the event in question is (e.g., a reel spin on a slot machine might last 3 s) (Parke and Griffiths, 2006, 2007). Here, it is important to note that duration of the betting/gambling event is different from event frequency (although they may be inextricably linked in so much as the length of a betting event will obviously limit the frequency with which they can take place). As Parke and Griffiths (2007) note, a betting event lasting 2 h (e.g., a soccer game) could not have an event frequency greater than one in any 2-h period but could have a betting frequency of over 100 with the advent of in-play betting (Griffiths, 2012).

In-play betting and gambling refers to the wagering on an event that has started but has not yet finished. This means gamblers can continue to bet on an event (e.g., a soccer or cricket match) and perhaps more importantly, adapt their bets according to how the event is progressing. For instance, in the UK, during the playing of almost any soccer match, a gambler can bet on everything from who is going to score the first goal, what the score will be after 30 min of play, how many yellow cards will be given during the game and/or in what minute of the second half will the first free kick be awarded (Griffiths, 2012). What the “in-play” gambling activities have done is take what was traditionally a discontinuous form of gambling—where a gambler made one bet every weekend on the result of the game—to one where a player can gamble continuously again and again (Griffiths, 2012). In short, the same game has been turned from what was a low event frequency gambling activity into a potentially high frequency one (and gone from an activity that had little association with problem gambling to one where problem gambling is far more likely among excessive in-play gamblers).

## Event duration and in-play gambling

Parke and Griffiths (2007) speculated that in-play betting has the potential to contribute to excessive, prolonged, unplanned, problem, and/or pathological betting and gambling as a result of: (1) within-session chasing on the same event or series of events (for example, an individual may make an incorrect bet selection, but then choose to recoup past losses by placing more bets on the same game; (2) an increase in perceived skill (through watching, analyzing, or even attending a betting event); and (3) simply making sporting events more exciting and/or interesting.

## Payout interval

Parke and Griffiths (2007) also noted another important (and related) structural characteristic—payout interval. This is the time between the end of the betting event (i.e., the outcome of the gamble) and the winning payment (if there is one). The frequency of playing when linked with two other factors—the result of the gamble (win or loss) and the actual time until winnings are received—exploits the psychological principles of learning. This process of operant conditioning conditions habits by rewarding (i.e., reinforcing) behavior (i.e., through presentation of a reward such as money). To produce high rates of response, those schedules which present rewards intermittently (random and variable ratio schedules) have shown to be most effective (Skinner, 1953).

Since a number of gambling activities (most notably slot machines) operate on random and variable ratio schedules it is unsurprising that excessive gambling can occur. Cornish (1978) noted that gaming operators appear to acknowledge the need to pay out winnings as quickly as possible thus indicating that receiving winnings is seen by the gaming industry to act as a reinforcement to winners to continue gambling. Rapid event frequency and short event duration also mean that the loss period is brief with little time given over to financial considerations and, more importantly, winnings can be re-gambled almost immediately (Parke and Griffiths, 2007). Games that offer a fast, arousing span of play, frequent wins, and the opportunity for rapid replay are those most associated with problem and pathological gambling (Griffiths, 2008). These parameters are structural and could theoretically be introduced into any gambling game. However, these structural characteristics alone do not always lead to habitual and/or addictive behavior. For instance, scratchcards have a potentially high event frequency, and a short gap between gambling and finding out the result of the gamble (Griffiths, 2000), but have a low addictive potential (DeFuentes-Merillas et al., 2003). Therefore, other structural characteristics may be important such as when the person actually receives the winning payment, which is not often immediate as scratchcard gamblers often gamble on the scratchcards at a different place to where they bought them, therefore the payout interval can be minutes, hours, or even days.

Clearly, money is a reward. However, gamblers may win in the short run but the vast majority of gamblers will eventually lose in the long run. It has also been pointed out that another potential reinforcer is activation—“the thrill of gambling” (Lea et al., 1987), and that this could play a role in all gambling situations. It may also play a role in mood modification and regulation. As Griffiths (1999) has noted:

“*There are also social rewards (e.g. raising of self-esteem, peer praise, social meaning of the activity, rites of passage, etc.). Further to this, Dickerson (1984) notes that there are multiple stimuli which can be perceived to be rewarding in gambling settings. Events such as the pre-race and race sequence at the race track, the spinning roulette wheel and the placing of bets can be reinforcing be cause they produce excitement, arousal and tension*”(p. 441).

There is also recent biological evidence of the potential reward value of gambling itself during the betting and anticipation phase of gambling. For instance, neuroimaging studies of the brain have shown that the expectation of a potential win, risky bets, and gambles are rewarding, and more so for a problem gambler than for a non-problem gambler (Miedl et al., 2012; Power et al., 2012; van Holst et al., 2012).

## The irrelevance of game type

The following two examples highlight the irrelevancy of game type and demonstrate that it is the structural characteristics rather than the game type that is critical in the acquisition, development, and maintenance of problem and pathological gambling for those who are vulnerable and/or susceptible. A “safe” slot machine could be designed in which no-one would ever develop a gambling problem. The simplest way to do this would be to ensure that whoever was playing the machine could not press the “play button” or pull the lever more than once a week. An enforced structural characteristic of an event frequency of once a week would almost guarantee that players could not develop a gambling problem. Alternatively, a problematic form of lottery could be designed where instead of the draw taking place weekly, bi-weekly, or daily, it would be designed to take place once every few minutes. Such an example is not hypothetical and resembles lottery games that already exist in the form of rapid-draw lottery games like keno (Griffiths and Wood, 2001).

Additionally, a recent study examining types of gambling and level of gambling involvement (using data from the 2007 British Gambling Prevalence Survey) indicated that when level of gambling is accounted, no specific type of gambling was associated anymore with disordered gambling, and that level of involvement in gambling better characterizes problem gambling (LaPlante et al., 2011).

## Conclusions

This short paper has argued that the frequency of opportunities to gamble (i.e., event frequency) when combined with other speed and frequency structural characteristics, appears to be a major contributory factor in the development of gambling problems and gambling pathology (Griffiths, 2008). The general rule is that the higher the event frequency, the more likely it is that the gambling activity will cause problems for the individual (particularly if the individual is susceptible and vulnerable). Problem and pathological gambling are essentially about rewards, and the speed and frequency of those rewards. Almost any game could be designed to either have high event frequencies or low event frequencies. Therefore, the more potential rewards there are, the more problematic and addictive an activity is likely to be and this is irrespective of game type as games such as diverse as lotteries and slot machines could have identical event frequencies and event durations. Given the time, money, and resources, a vast majority of gambling activities are “continuous” in that people have the potential to gamble again and again and this is also one of the factors that may contribute to the acquisition, development, and maintenance of problem and pathological gambling.
